# Acknowledgment to Reviewers of *Pharmacy* in 2021

**DOI:** 10.3390/pharmacy10010021

**Published:** 2022-01-27

**Authors:** Pharmacy Editorial Office

**Affiliations:** MDPI AG, St. Alban-Anlage 66, 4052 Basel, Switzerland

Rigorous peer-reviews are the basis of high-quality academic publishing. Thanks to the great efforts of our reviewers, *Pharmacy* was able to maintain its standards for the high quality of its published papers. Thanks to the contribution of our reviewers, in 2021, the median time to first decision was 18 days and the median time to publication was 42 days. The editors would like to extend their gratitude and recognition to the following reviewers for their precious time and dedication, regardless of whether the papers they reviewed were finally published:
Abir B. KaramiJialin ZhangAfonso M. CavacoJin Young NamAkshaya BhagavathulaJinming HuAlba Marcos-DelgadoJinxin ZhaoAleda ChenJoanne FullerAleš ObrezaJoel NitzkinAlexey MelnikovJóhannes ReynissonAllison ParsonsJohn C. HaydenAlly Dering AndersonJohn HawboldtAmadeus DobrescuJohn Mark FroilandAmanda MixonJon P. WietholterAmanda R MargolisJordan R. CovveyAmira GuirguisJoy SparkAmy L. ShaverJuan Manuel MendiveAna PerisinJulie MasonAnandi LawJustyna RójAndrea ManfrinKaining ZhiAndrey GlotovKaren WhitfieldAndries KosterKari L. OlsonAnita MajchrowskaKarin SvensbergAnna Birna AlmarsdottirKaryn M. SullivanAntonio MastinoKatarzyna Ptaszynska-KopczynskaAntonio Sánchez PozoKatelynn MayberryArijana MestrovicKatherine K. KnappArit Esio UdohKathryn SteadmanArtur Cieslar-PobudaKelly GrantArtur OwczarekKenneth LeeAvi PeretzKerry MansellAvinash KolliparaKevin CoulouresBarry BleskeKevin FujiBee Kang TanKevin SneedBela KocsisKiriaki KeramitsoglouBenoit AllenetKirstie GalbraithBernhard LangerKonstantinos KontogiannopoulosBetty ExintarisKota KodamaBeverly FitzpatrickKreshnik HotiBoohwi HongKristin JankeBorut BožičKristina GervinBradley J. LangfordKuan-Hsuan ChenBrent RollinsKurt E. HersbergerBrett ToelleL Douglas RiedBrian PiperLana MinshewBrian QuayLilian AzzopardiBruce SunderlandLiliana RogozeaCarla Maria Batista Ferreira PiresLingpeng ShanCarla PiresLiz BreenCarlos Llanes-ÁlvarezLong Chiau MingCarlos Mauricio BarbosaLorinda AndersonCarlotta LunghiLouise S. DeeksCarol NashLuca PaluganCatriona BradleyLyn Li LimCharlene R. WilliamsMainul HaqueChiau Ming LongMaja MeskoChristian CavéMaja Ortner HadžiabdićChristian MesengeMajdi N. Al-HasanChristina SakarikouMarc SimardChristopher OwensMarc SturgillClaire MannMarcello SariniClare GuildingMaree SimpsonClaudia AltamuraMaresca Attard PizzutoClémence PerraudinMaria Angeles Caballero-MoraConxita MestresMaria BagattiniConxita Mestres MirallesMaria D DonovanCorina NagyMaria Rosa Suñer SolerCourtney E. GamstonMarie BarnardDaisy VolmerMariola DrozdDalia Al MaghaslahMark NauntonDana A. BrownMartina BientzleDanguolė RugytėMary Beth O’ConnellDaniel Echeverria-EsnalMary BushellDanuta GajewskaMary-Claire KennedyDario FerreroMaya AzradDarko ModunMeagen RosenthalDavid H. KrelingMegan SmithDavid P. ZgarrickMeghan JeffersDavid R. SteebMelody RyanDavid SteebMicha M.M. WilhelmusDawit Teshome GebregeorgiseMichael BeazelyDean ElbeMichael TwiggDelia Mirela TitMichela GrossoDennis M. WilliamsMichele PergadiaDerek P. EvansMichelle FloodDhan ShresthaMiha SkvarčDiana LupuMikael J. HoffmannDiane Ashiru-OredopeMike ScottDiptiman BoseMing Jie LeeDominik StämpfliMircea Ioan PopaDoris RusicMohammed S SalahudeenDuncan HillMohiuddin AhmedElizabeth UnniMojtaba VaismoradiEllen CarlNatalie CrownElsa López-PintorNazareth Martínez-HerediaErin HolmesNejc HorvatErshuai ZhangNicole Cieri-HutchersonEwa Gibuła-TarłowskaNikolas A. S. ChottaF. Javier Del RíoNilesh PatelFátima RoqueNorman Fenn IIIFerdinando Luca LoriniOlajide O. FadareFrank RomanelliOluwafemi AdeagboFrank SanfilippoOuti SalminenFritzie S. AlbarilloP. Brandon BookstaverGeorgina Silva-SuárezParastou DonyaiGeorgios DinosPascal Le CorreGiana Carli LorenziniPatricia LoganGianni LazzarinPaulina CorralGiovanna RicciPaulina StehlikGiovanni DamianiPauline DouglasGiovanni SignorePeter MizseyGiuseppina MalcangiPetra ThürmannGregory PetersonPhil AyersGuilherme MontesPhilippe Morency-PotvinGuillaume HachePhu DuongGustavo PimentelPilar ModamioHae Kyung LeePilar Pérez-RosHamde NazarPiotr MerksHashem Salarzadeh JenatabadiPiotr PoznańskiHomaira HanifRafael BaptistaHugo SarmentoRǎzvan PetcaHyunah ChoRenee RobinsonIan HeslopRenke MaasImti ChoonaraRenzo ZanottiIsabelle PrêcheurRia BenkőIulia-Cristina BagiuRichard ParrishIvan BindoffRita AgarwalJacqueline E. McLaughlinRobert IgnoffoJaekyu ShinRohan A. ElliottJames EdwardsRoopma WadhwaJames W. FettermanRucha BondJamie L. WagnerSamantha HollingworthJanka PetravićShigeo YamamuraJason PerpelkinSurya Prakasarao KovvasuJean MoonVictoria BelousovaJeff CainYoshihiro NoguchiJeff TaylorZhe HanJessica Thompson



